# Indexes of ferroptosis and iron metabolism were associated with the severity of diabetic nephropathy in patients with type 2 diabetes mellitus: a cross-sectional study

**DOI:** 10.3389/fendo.2023.1297166

**Published:** 2023-12-22

**Authors:** Pingping Zhao, Xiaoyu Lv, Zhicong Zhou, Xiaolan Yang, Ying Huang, Jingfang Liu

**Affiliations:** ^1^ The First Clinical Medical College, Lanzhou University, Lanzhou, Gansu, China; ^2^ Kangle Dental Clinic, Baiyin, Gansu, China; ^3^ Clinical Laboratory of The First People’s Hospital of Baiyin, Baiyin, Gansu, China; ^4^ Department of Endocrinology, The First Hospital of Lanzhou University, Lanzhou, Gansu, China

**Keywords:** type 2 diabetes, ferroptosis, GPx4, ACSL4, iron metabolism

## Abstract

**Objective:**

To explore the correlations between diabetic nephropathy (DN) and serum levels of glutathione peroxidase 4 (GPX4), acyl-CoA synthetase long-chain family member 4 (ACSL4), iron, transferrin (Tf), and ferritin in patients with type 2 diabetes mellitus (T2DM).

**Methods:**

According to the urinary albumin excretion rate(UAER) or estimated glomerular filtration rate (eGFR) levels, a total of 123 patients with T2DM were separately divided into normoalbuminuria (NO), microalbuminuria (MI), macroalbuminuria (MA) groups, and G1 (eGFR ≥ 90 mL/min), G2 (eGFR ≤ 60 mL/min to < 90 mL/min), and G3 groups (eGFR< 60 mL/min), with 33 healthy participants as the control (HC). The differences in serum GPX4, ACSL4, iron, Tf, and ferritin levels between groups were compared, and the relationships between these levels were analysed. The independent correlations between UAER or DN severity and serum GPX4, ACSL4, iron, Tf, and ferritin levels were analysed by multiple linear and multinomial logistic regression, respectively.

**Results:**

To the patients with T2DM, with the increase in UAER levels, GPX4, iron, and Tf levels gradually decreased, whereas ACSL4 levels increased, meanwhile with the decrease in eGFR levels, GPX4 and Tf levels gradually decreased, whereas ACSL4 levels increased. UAER were independently and positively correlated with ACSL4 [β = 17.53, 95% confidence interval (CI; 11.94, 23.13)] and negatively correlated with GPX4 [β = −1.633, 95% CI (−2.77, -0.496)] and Tf [β = −52.94, 95% CI (-95.78, −10.11)].The NO and MI groups were considered as reference groups, respectively. The severity of DN was negatively correlated with serum GPX4 [odds ratio (OR) = 0.925 and 0.902, *p =*0.015 and 0.001], and Tf (OR = 0.109 and 0.119, *p =*0.043 and 0.034), and positively correlated with ACSL4 (OR = 1.952 and 1.865, both *p <*0.001) in the MA group.

**Conclusion:**

DN severity was negatively correlated with serum GPX4 and Tf levels and positively correlated with serum ACSL4 levels in patients with T2DM.

## 1 Introduction

Diabetes is a chronic disease that seriously affects human health. In the last 20 years, the number of people with diabetes worldwide has more than doubled (1). Diabetic nephropathy (DN), as one of the most common microvascular complications of diabetes and the main cause of end-stage renal disease (ERSD), is closely related to the increased mortality of diabetic patients, and about 40% of diabetic patients eventually develop DN (2). Early diagnosis and treatment of DN can prevent the further deterioration of renal function in diabetic patients (3, 4). There are many pathways and mediators involved in the development and progression of DN, including oxidative stress, angiotensin II (Ang-II), and inflammatory processes, which are recently considered to play an important role (5). Currently, the treatment methods for DN mainly include controlling blood pressure and blood sugar and using angiotensin-converting enzyme inhibitors, etc. However, the above methods have limited effect on preventing the progression of DN (6). Therefore, it is necessary to conduct in-depth research on the pathogenesis of DN to explore new methods of diagnosis and treatment.

Iron metabolism is the process by which iron is absorbed, transported, distributed, stored, utilised, transformed, and excreted by an organism. It has been demonstrated that iron overload induces oxidative stress and promotes kidney damage in diabetic rats (7).Previous studies have found that the increased iron deposition in the kidneys of T2DM patients progressing to DN (8) as well as a connection between serum ferritin and urinary microalbumin (9). However, in-depth research on the relationship between DN and serum iron, transferrin (Tf), and ferritin is lacking.

Ferroptosis, an iron-dependent, non-apoptotic type of cell death, is characterised by iron overload, the accumulation of reactive oxygen species (ROS), and lipid peroxidation (10). Over the past few years, the process and function of ferroptosis have been well studied. Its main regulators include systemic Xc-, glutathione peroxidase 4 (GPX4), p53, ferroptosis suppressor protein 1, acyl-CoA synthetase long-chain family member 4 (ACSL4), and nuclear factor erythroid 2-related factor (Nrf-2) (10). GPX4 can convert the peroxide bond of lipid peroxidation (L-OOH) to a hydroxyl group (L-OH) by the action of the cofactor glutathione, and loses its peroxide activity (11). This process counteracts ferroptosis in cells. ACSL4 participates in the synthesis of polyunsaturated fatty acid-containing phospholipids that can form lipid hydroperoxides via the action of lipoxygenase, which can promote cellular ferroptosis (12).

ROS accumulation is at the centre of ferroptosis (13). In addition, multiple ROS production pathways have been identified as potentially major factors in the pathogenesis of DN (14), suggesting that ferroptosis may be related to DN. The mechanisms underlying ferroptosis in DN were initially studied primarily at the cellular and animal levels. Wang et al. found that in a DN mouse model, the expression of the ferroptosis-related protein GPX4 decreased, whereas the expression of ACSL4, and the lipid peroxide products and iron content increased (15). Subsequently, Kim et al. reported that the ferroptosis-related molecules SlC7A11 and GPX4 were reduced in kidney biopsy samples from patients with DN compared to those from patients without DN (16).These studies indicate that ferroptosis is associated with DN.

As mentioned above, GPX4 and ACSL4 are key regulators of ferroptosis process; while Tf and ferritin reflect the transport, binding and storage of iron in the body, respectively, and are the core molecules to maintain the balance of iron metabolism in the body. Therefore, in this study, we compared serum iron, Tf, ferritin, GPX4, and ACSL4 levels in T2DM patients with and without DN to analyse the correlation between DN and ferroptosis or iron metabolism.

## 2 Participants and methods

### 2.1 Participants

**Figure 1:**
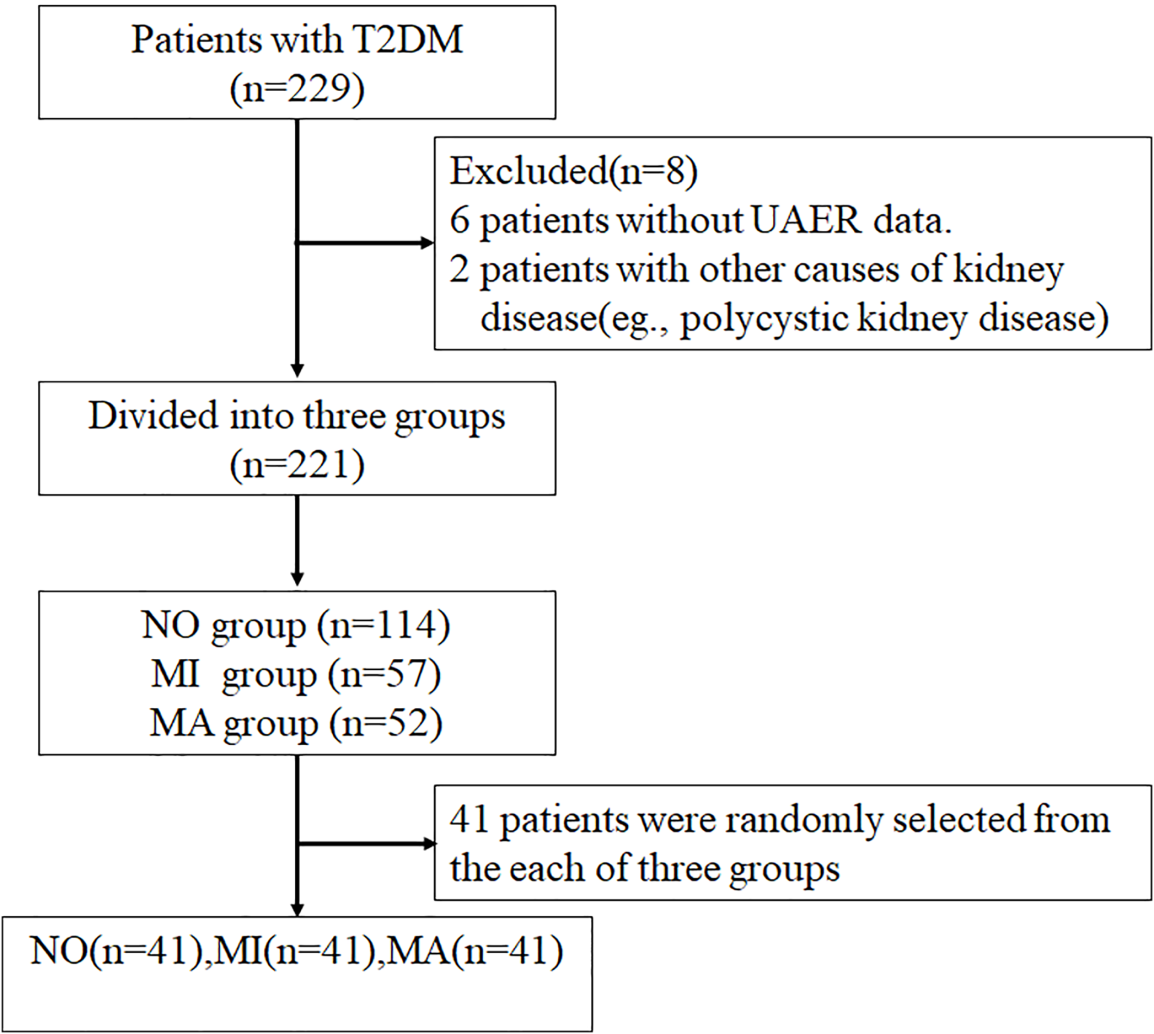
Diagram of the study design.

In total, 229 patients with T2DM admitted to the Department of Endocrinology, The First Hospital of Lanzhou University from October 2019 to December 2020 were enrolled in this study. Of these, 221 patients were screened according to the inclusion and exclusion criteria and divided into three groups: the normoalbuminuria group [NO, urinary albumin excretion rate (UAER)<20 µg/min)] with 113 patients, the microalbuminuria group [MI, 20 ≤ UAER<200 µg/min] with 57 patients, and the macroalbuminuria group [MA, UAER ≥ 200 µg/min] with 51 patients. Forty-one patients were randomly selected from each of these three groups by simple random sampling using Statistical Package for the Social Sciences (version 26.0; IMB, Armonk, New York, USA). Ultimately, 123 patients were enrolled in this study, and 33 healthy participants were included as controls. This study was approved by the Ethics Committee of The First Hospital of Lanzhou University. A STROBE flowchart is shown in **Figure 1**.

Sample size estimation

Serum GPX4, ACSL4, iron, Tf, and ferritin levels were used as the main outcome indexes for sample size calculation, and the ratio of sample size for each group was 1:1:1:1. We used Power Analysis and Sample Size (PASS, version 15.0) for the calculations, setting testing level α =0.05, testing effectiveness β =0.1 (17), the estimates of the mean and standard deviation for each group were obtained using the results of the pre-experiment, and the number of included cases in each group was estimated to be at least 29.

#### 2.1.1 Inclusion criteria

(1) Patients with T2DM aged ≥ 18 years; (2) Duration of T2DM ≥1 year; (3) T2DM diagnosed according to the 1999 World Health Organization criteria;(4) DN was defined as patients with T2DM and UAER ≥ 200µg/min or patients with diabetes and 20≤ UAER<200µg/min and/or estimated glomerular filtration rate (eGFR) less than 60ml/min/1.73m2 (18).

#### 2.1.2 Exclusion criteria

(1) Type 1 diabetes, gestational diabetes, or other special types of diabetes; (2) Acute complications of diabetes, such as diabetic ketoacidosis and hyperosmolar hyperglycaemia; (3) Kidney diseases caused by diseases other than diabetes, such as primary nephrotic syndrome, glomerulonephritis, renal artery stenosis, acute renal failure, polycystic kidney disease, and urinary tract infection; (4) Lack of clinical data.

### 2.2 Methods

#### 2.2.1 Data collection

##### 2.2.1.1 Characteristics of the participants

The name, sex, age, height, weight, systolic and diastolic blood pressures (SBP and DBP, respectively), diabetes duration, hypertension (HP), and history of other diseases and therapeutic drugs prescribed to each participant were reviewed.

The Body mass index (BMI) was calculated as weight (kg) divided by height (m^2^).

##### 2.2.1.2 Laboratory examination

The levels of total cholesterol (TC), triglycerides (TG), high-density lipoprotein cholesterol (HDL-C), low-density lipoprotein cholesterol (LDL-C), haemoglobin (Hb) serum uric acid (UA), serum creatinine (SCr), and iron were measured using a BS-220 automatic biochemical analyser (Shenzhen Mindray Bio-Medical Electronics Co., Ltd., Shenzhen, China). Glycosylated haemoglobin (HbAlc) levels were measured via high-performance liquid chromatography (Bio-Rad-D10; Bio-Rad Laboratories, Hercules, California, USA). Fasting plasma glucose (FPG) and fasting insulin (FINS) levels were measured using chemiluminescence assays (ADVIA Centaur-XP; Siemens Healthineers, Erlangen, Germany). Urinary microalbumin levels were determined using rate-scatter turbidimetry, and urinary creatinine (CR) was measured using the creatinine oxidase method.

Serum ferritin levels were determined using an electrochemiluminescence immunoassay (Roche, Shanghai, China).Serum Tf levels were determined viaimmuno-turbidimetry (Hitachi), the linear range was 0.05– 6.8 g/L, the intra-batch relative deviation was ≤6.0%, and the inter-batch relative range was ≤10.0%.

Serum GPX4 levels were determined via an enzyme-linked immunosorbent assay (HM11433, Bioswamp, Wuhan, China); the detection range was 0.75 – 60 ng/ml, the sensitivity was ≤0.15 ng/ml, and the intra-plate and inter-plate coefficients of variation were ≤9% and ≤11%, respectively. Serum ACSL4 levels were determined using an enzyme-linked immunosorbent assay (HM13015, Bioswamp, Wuhan, China); the detection range was 0.125 – 10 ng/ml, the sensitivity was ≤0.025 ng/ml, and the intra-plate and inter-plate coefficients of variation were ≤9% and ≤11%, respectively.

Homeostasis model assessment of insulin resistance (HOMA-IR) was performed using the following formula: HOMA-IR = FPG × FINS/22.5.

eGFR was calculated using the following formula: eGFR = 186 × SCr^-1.154^ × age^-0.203^ (Female × 0.742) mL/min/1.73 (19).

#### 2.2.2 Statistical methods

Data were analysed using Statistical Package for the Social Sciences (version 26.0; IMB, Armonk, New York, USA). Normally distributed measurement data were expressed as the mean ± standard deviation ( $\bar{x}$ *± s*), and non-normally distributed measurement data were expressed as the median and interquartile range. Numerical data are expressed as frequencies and percentages (%). According to the normal or non-normal distributions of measurement data, one-way ANOVA and the Kruskal-Wallis test were used for comparisons among groups, whereas the chi-square test was used for analysing numerical data. The *p* values of intragroup multiple comparisons were adjusted using the Bonferroni method. Correlation analysis was performed using Pearson’s or Spearman’s rank correlation tests. Multiple linear regression was used to evaluate the independent correlations between UAER and serum GPX4, ACSL4, iron, Tf, and ferritin levels. Associations between DN severity and iron metabolism or ferroptosis-related indexes were assessed using multinomial logistic regression. *P* < 0.05 was considered statistically significant.

## 3 Results

### 3.1 Clinical characteristics of study participants

In total, 123 patients with T2DM (79 males and 44 females) were included in this study, with 33 healthy participants as controls.

Age, SBP, DBP, FPG, FINS, HOMA-IR, and TG levels in the NO, MI, and MA groups were higher (all *p* < 0.05), whereas HDL-C levels were lower in T2DM patients (*p* < 0.05) than those in the HC group. The UA level was higher in the MI group than that in the HC group (*p* < 0.05), and also higher in the MA group than those in the HC, NO, and MI groups (all *p* < 0.05).

SCr levels were higher in the MI group than those in the HC and NO groups (both *p* < 0.05), and also higher in the MA group than those in the HC, NO, and MI groups (all *p* < 0.05). The eGFR was lower in the MI group than those in the HC and NO groups (both *p* < 0.05), and also lower in the MA group than those in the HC and NO groups (both *p* < 0.05) (**Table 1**).

**Table 1:** Clinical characteristics of study participants.

Index	HC (33)	NO (41)	MI (41)	MA (41)	*P*
Female(%) Male(%)	25(75.8) 8(24.2)	12(29.3) 29(70.7)*	20(48.8) 21(51.2)*	12(29.3) 29(70.7)*	<0.001
Age(years)	41.94 ± 13.90	60.27 ± 9.20**	64.41 ± 7.93**	61.05 ± 10.03**	<0.001
SDP(mmHg)	114.58 ± 10.43	139.90 ± 16.95**	148.10 ± 17.48**	147.51 ± 18.54**	<0.001
DBP(mmHg)	67.70 ± 6.83	80.44 ± 11.25**	80.49 ± 11.81**	80.83 ± 11.84**	<0.001
BMI(kg/m^2^)	23.28 ± 3.72	25.06 ± 2.82	24.69 ± 3.16	24.69 ± 2.85	0.089
FPG(mmol/L)	4.59 ± 0.35	9.43 ± 3.46**	9.71 ± 3.44**	9.46 ± 2.71**	<0.001
FINS(mU/L)	3.10(2.51,4.38)	7.18(5.30,9.58)**	8.06(4.81,11.49)**	8.49(5.82,13.34)**	<0.001
HOMA-IR	0.63(0.49,0.90)	2.91(1.79,4.27)**	3.86(2.48,5.21)**	3.04(1.98,7.42)**	<0.001
HbAlc(%)	––––––––––––––	8.40 ± 2.00	9.20 ± 1.90	8.78 ± 1.77	0.167
Hb(g/L)	118.58 ± 23.94	117.95 ± 17.91	123.59 ± 19.34	112.85 ± 15.36	0.095
TC(mmol/L)	4.21 ± .52	4.12 ± 0.92	4.33 ± 1.05	4.68 ± 1.95	0.2
TG(mmol/L)	0.97(0.66,1.22)	1.43(1.02,1.95) *	1.82(1.35,2.40)**	1.90(1.23,2.87)**	<0.001
HDL-C(mmol/L)	1.34 ± 0.22	1.04 ± 0.30*	1.01 ± 0.20**	1.06 ± 0.48*	<0.001
LDL-C(mmol/L)	2.72 ± 0.45	2.82 ± 0.76	2.77 ± 0.71	3.05 ± 1.28	0.371
UA(μmol/L)	288.39 ± 72.03	315.39 ± 77.57	343.68 ± 75.77*	401.12 ± 83.56**^##$^	<0.001
SCr(μmol/L)	65.55 ± 10.85	62.32 ± 12.02	82.12 ± 26.47*^#^	105.41 ± 40.83**^##$^	<0.001
eGFR(ml/min)	101.75 ± 15.40	115.73 ± 25.01	82.47 ± 23.80*^##^	72.37 ± 33.36**^##^	<0.001
UAER(μg/min)	––––––––––––––	13.00(10.00,15.70)	72.90(56.00,117.16) **^##^**	286.40(244.30,390.75) **^##$$^**	<0.001

SBP, systolic blood pressure; DBP, diastolic blood pressure; BMI,:body mass index; FPG, Fasting Plasma Glucose; FINS, fasting insulin; HOMA-IR, homeostasis model assessment of insulin resistance; HbAlc, glycosylated hemoglobin; Hb, hemoglobin; TC, total cholesterol; TG, triglycerides; HDL-C, high-density lipoprotein cholesterol; LDL-C, low-density lipoprotein cholesterol; UA, serum uric acid; SCr, serum creatinine; eGFR, estimated glomerular filtration rate; Tf, transferrin; UAER, urinary albumin excretion rate. DR, diabetic retinopathy; Compared with HC group, *p<0.05, **p<0.001; Compared with NO group **^#^** p<0.05, **^##^** p<0.001; Compared with MI group, **^$^** p<0.05, **^$$^** p<0.001.

### 3.2 Comparison of serum levels of GPX4, ACSL4, and iron metabolism-related indexes in the T2DM groups with different urinary albumin levels

**Figure 2:**
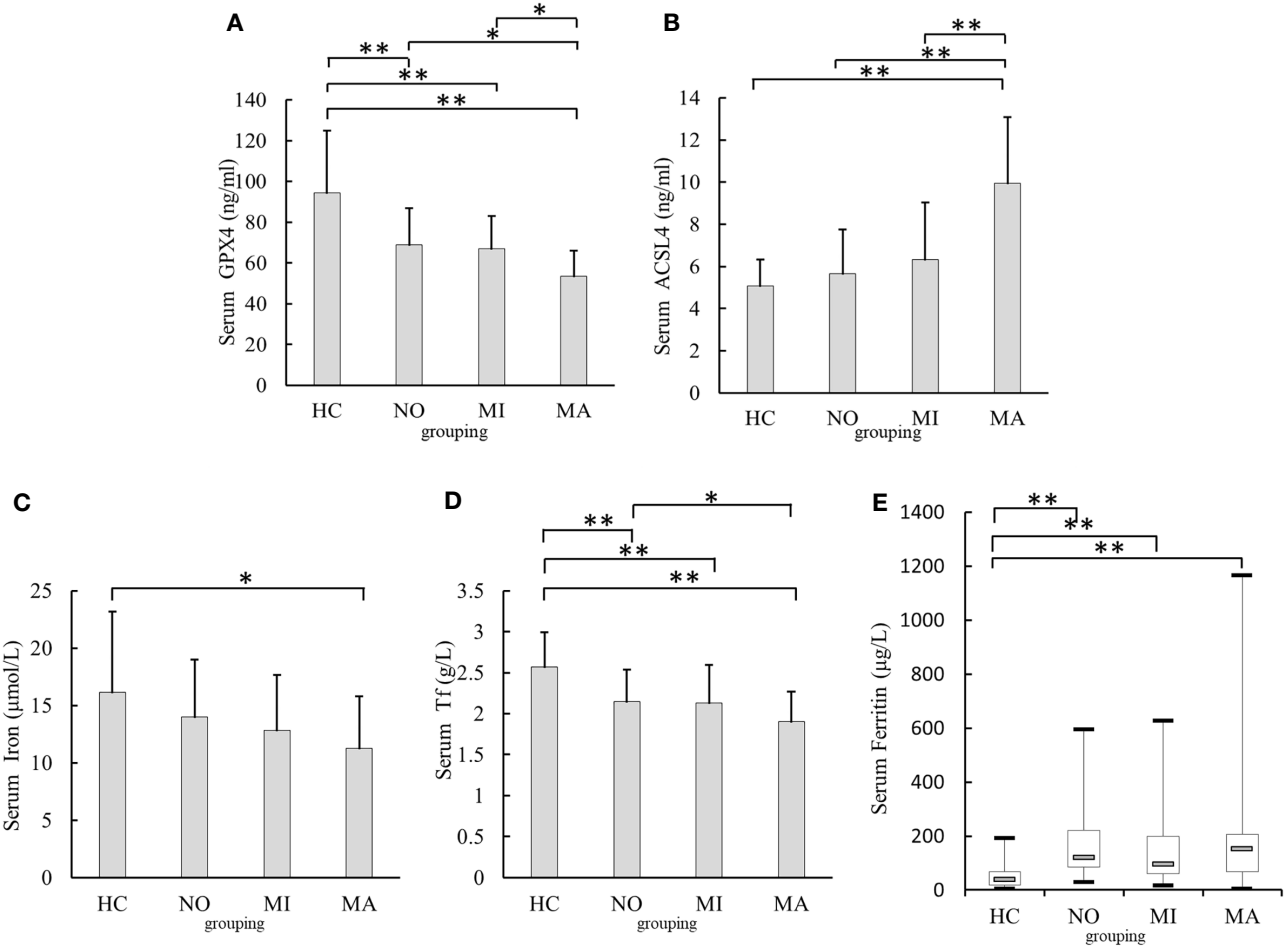
Comparison of serum GPX4, ACSL4,iron,Tf, and Ferritin levels in different urinary albumin levels groups. **(A)** Comparison of serum GPX4 levels. **(B)** Comparison of serum ACSL4 levels. **(C)** Comparison of serum iron levels. **(D)** Comparison of serum Tf levels. **(E)** Comparison of serum Ferritin levels. NO, normoalbuminuria group; urinary albumin excretion rate(UAER)<20μg/min; MI, microalbuminuria group:20μg/min≤UAER<200μg/min; MA, macroalbuminuriagroup,UAER≥200μg/min; GPX4, glutathione peroxidase 4; ACSL4, acyl-CoA synthetase long-chain family member 4; Tf, transferrin;*p<0.05;**p<0.001.

Serum GPX4 levels were lower in the T2DM with MA [(53.54 ± 12.55)ng/ml, *p <*0.001], MI[(67.05 ± 16.07)ng/ml, *p <*0.001], and NO groups [(68.97 ± 17.95)ng/ml, *p <*0.001] than in the HC group [(94.35± 30.71)ng/ml]. In addition, serum GPX4 levels in the MA group were significantly lower than those in the MI and NO groups (*p*= 0.014, *p*= 0.003) (**Figure 2A**). Serum ACSL4 level in the MA group was higher [(9.96 ± 2.84)ng/ml, *p<*0.001]than that in the HC group [(5.07± 1.26)ng/ml].In addition, serum ACSL4 levels in MA group were higher than those in the NO and MI groups [(5.67 ± 2.11)ng/ml and [(6.31 ± 2.72)ng/ml, respectively; both *p <*0.001](**Figure 2B**). Serum iron level in the MA group [(11.26 ± 4.55)μmol/l] was lower than that in the HC group [(16.12 ± 7.07)μmol/l, *p* = 0.001](**Figure 2C**). Serum Tf levels in the NO [(2.15 ± 0.39)g/l],MI [(2.13 ± 0.46)g/l], and MA groups[(1.90 ± 0.37)g/l]were lower than those in the HC group [(2.57 ± 0.42)g/l, all *p* < 0.001]Serum Tf level in the MA group was lower than that in the NO group (*p* = 0.047) (**Figure 2D**). Serum ferritin levels in the NO [(120.55(84.76-220.44)μg/l], MI[(97.00(59.51 –198.25)μg/l], and MA groups [(153.49(66.95 – 206.92)μg/l] were higher than those in the HC group [38.30(18.40 – 68.00)μg/l, all *p*< 0.001](**Figure 2E**)

### 3.3 Comparison of serum levels of GPX4, ACSL4, and iron metabolism-related indexes in the T2DM groups with different eGFR levels

Patients with T2DM were divided into three groups according to eGFR levels: G1 (eGFR≥ 90 mL/min), G2 (eGFR ≤ 60 mL/min to < 90 mL/min), and G3 (eGFR< 60 mL/min), with healthy participants as the HC group.

**Figure 3:**
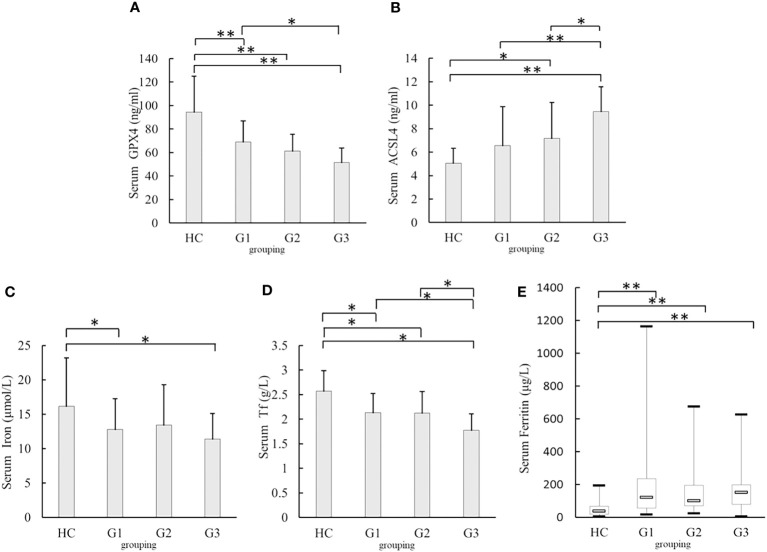
Comparison of serum GPX4, ACSL4, iron, Tf, and Ferritin levels in different eGFR levels **(A)** Comparison of serum GPX4 levels. **(B)** Comparison of serum ACSL4 levels. **(C)** Comparison of serum iron levels. **(D)** Comparison of serum Tf levels. **(E)** Comparison of serum Ferritin levels. G1:estimated glomerular filtration rate(eGFR)≥ 90 mL/min, G2:eGFR≤ 60 mL/min to < 90 mL/min;G3:eGFR< 60 mL/min; HC, healthy participants; GPX4, glutathione peroxidase 4; ACSL4, acyl-CoA synthetase long-chain family member 4; Tf, transferrin*p<0.05;**p<0.001.

The serum GPX4 levels in the G3 [(51.55 ± 12.16)ng/ml], G2[(61.27 ± 14.39)ng/ml], and G1 groups[(69.19 ± 17.8)ng/ml]were lower than those in the HC group [(94.35 ± 30.71)ng/ml, all *p <*0.001]. Serum GPX4 level in the G3 group was lower than that in the G1 group (*p*= 0.003) (**Figure 3A**). Serum ACSL4 levels in the G3 group [(9.45 ± 2.12)ng/ml] were higher than those in the HC [(5.07 ± 1.26)ng/ml, *p <*0.001], G1 [(6.57 ± 3.31)ng/ml, *p <*0.001]and G2 groups [(7.18 ± 3.04)ng/ml, *p =*0.011] The serum ACSL4 level in the G2 group [(7.18 ± 3.04)ng/ml]was higher than that in the HC group [(5.07 ± 1.26)ng/ml, *p =*0.007](**Figure 3B**). The serum iron levels in the G1 and G3 groups [(12.69 ± 4.45)μmol/l and (11.37 ± 3.74)μmol/l, respectively] were lower than those in the HC group [(16.12 ± 7.07)μmol/l, *p =* 0.025 and 0.009) (**Figure 3C**). The serum Tf levels in the G1 [(2.13 ± 0.39)g/l], G2 [(2.12 ± 0.44)g/l], and G3groups[(1.77 ± 0.34)g/l]were lower than those in the HC group [(2.57 ± 0.42)g/l, all *p* < 0.001], and serum Tf levels in the G3 group were lower than those in the G1 and G2 groups (*p* =0.002 and 0.005) (**Figure 3D**). Serum ferritin levels in the G1 [122.91(56.77 – 235.28)μg/l], G2 [102.27(70.60 – 196.54)μg/l], and G3 groups [153.49(79.62 – 2198.82)μg/l]were higher than those in the HC group [38.30(18.40 - 68.00)μg/l, all *p <*0.001) (**Figure 3E**).

### 3.4 Correlation between UAER or eGFR levels and serum GPX4, ACSL4, and iron metabolism indexes in patients with T2DM

Pearson’s correlation analysis showed that UAER levels were positively correlated with ferritin and ACSL4 levels (r = 0.187, *p* = 0.038; r=0.546, *p* < 0.001), and negatively correlated with Tf and GPX4 levels (r = −0.285, *p*= 0.001; r = −0.413, *p* < 0.001) (**Table 2**).

**Table 2:** Correlation between UAER or eGFR levels and serum GPX4, ACSL4, and iron metabolism indexes in patients with T2DM.

Index	UAER		eGFR	
	r	*P*	r	*P*
Sex(Male=1,Female=2)	-0.67	0.46	-0.126	0.117
Age	-0.28	0.755	-0.262	<0.001
SBP	0.12	0.187	-0.267	0.001
DBP	0.03	0.742	-0.109	0.175
BMI	-0.089	0.329	-0.023	0.78
FPG	-0.001	0.996	-0.143	0.075
FINS	0.217	0.016	-0.146	0.069
HOMA-IR	0.103	0.258	-0.085	0.293
HbAlc	0.076	0.404	-0.046	0.617
TC	0.159	0.079	-0.085	0.294
TG	0.186	0.039	-0.097	0.230
HDL	-0.013	0.883	-0.007	0.93
LDL	0.122	0.178	-0.031	0.698
UA	0.41	<0.001	-0.446	<0.001
SCr	0.568	<0.001	-0.824	<0.001
eGFR	-0.493	<0.001	–––––––––––	–––––––––––
Iron	-0.188	0.037	0.1	0.215
Tf	-0.285	0.001	0.243	0.002
Ferritin	0.187	0.038	-0.019	0.816
GPX4	-0.413	<0.001	0.302	<0.001
ACSL4	0.546	<0.001	-0.198	0.013
UAER	–––––––––––	–––––––––––	-0.493	<0.001

SBP, systolic blood pressure; DBP, diastolic blood pressure; BMI,:body mass index; FPG, Fasting Plasma Glucose; FINS, fasting insulin; HOMA-IR, homeostasis model assessment of insulin resistance; HbAlc, glycosylated hemoglobin; TC, total cholesterol; TG, triglycerides; HDL-C, high-density lipoprotein cholesterol; LDL-C, low-density lipoprotein cholesterol; UA, serum uric acid; SCr, serum creatinine; eGFR, estimated glomerular filtration rate; Tf:transferrin; GPX4:glutathione peroxidase 4; ACSL4, acyl-CoA synthetase long-chain family member 4; UAER, urinary albumin excretion rate.

eGFR levels were positively correlated with Tf and GPX4 levels (r = 0.243, *p* = 0.002; r = 0.302, *p* < 0.001), and negatively correlated with ACSL4 levels(r = −0.493, *p* < 0.001) (**Table 2**).

### 3.5 Simple and multiple linear regression analysis of the independent correlated factors of UAER levels in patients with T2DM

UAER was considered as the dependent variable, and sex, age, SBP, DBP, BMI, FPG, FINS HOMA-IR, HbAlc, TC, TG, HDL-C, LDL-C, UA, SCr, eGFR, iron, Tf, ferritin, GPX4, and ACSL4 as the independent variables. Simple linear regression analysis showed that UAER were positively correlated with FINS,TG, UA, SCr, ferritin, and ACSL4 (all *p*< 0.05) and negatively correlated with eGFR, iron, Tf, and GPX4 (all *p*< 0.05).

The UAER-correlated variables as independent variables, the multiple linear regression analysis was conducted through the stepwise method. The results showed that UAER levels were independently and positively correlated with UA, SCr, and ACSL4, and negatively correlated with Tf and GPX4 (**Table 3**).

**Table 3:** Simple and Multiple linear regression analysis of the independent correlated factors of UAER levels in patients with T2DM.

Index	B (95%CI)	t	*P*
Simple linear regression analysis			
Sex(Male=1,Female=2)			0.460
Age	-0.434(-3.187,2.318)	-0.312	0.755
SBP	0.940(-0.463,2.343)	1.326	0.187
DBP	0.365(-1.828,2.559)	0.330	0.742
BMI	-4.269(-12.89,4.353)	-0.980	0.329
FPG	-0.022(-7.949,7.904)	-0.006	0.996
FINS	2.763(0.525,5.001)	2.444	0.016
HOMA-IR	3.230(-2.397,8.856)	0.838	0.258
HbAlc	5.598(-7.627,18.824)	0.838	0.404
TC	16.054(-1.892,34.00)	1.771	0.079
TG	14.087(0.723,27.451)	2.087	0.039
HDL	-5.526(-79.49,68.44)	-0.148	0.883
LDL	18.07(-8.355,44.50)	1.354	0.178
UA	0.670(0.401,0.938)	4.945	<0.001
SCr	2.375(1.755,2.995)	7.582	<0.001
eGFR	-2.089(-2.753,-1.425)	-6.228	<0.001
Iron	-5.436(-10.54,-0.335)	-2.110	0.037
Tf	-95.15(-152.7,-37.56)	-3.271	0.001
Ferritin	0.167(0.009,0.325)	2.093	0.038
GPX4	-3.564(-4.907,-2.220)	-5.250	<0.001
ACSL4	24.09(17.43,30.76)	7.1600	<0.001
Multiple linear regression analysis			
UA	0.292(0.061,0.523)	2.504	0.014
SCr	1.060(0.405,1.714)	3.205	0.002
Tf	-52.94(-95.78,-10.11)	-2.448	0.016
GPX4	-1.633(-2.77,-0.496)	-2.845	0.005
ACSL4	17.53(11.94,23.13)	6.204	<0.001

UAER, urinary albumin excretion rate; SBP, systolic blood pressure; DBP, diastolic blood pressure; BMI, body mass index; FPG, Fasting Plasma Glucose; FINS, fasting insulin; HOMA-IR, homeostasis model assessment of insulin resistance; HbAlc, glycosylated hemoglobin; TC, total cholesterol; TG, triglycerides; HDL-C, high-density lipoprotein cholesterol; LDL-C, low-density lipoprotein cholesterol; UA, serum uric acid; SCr, serum creatinine; eGFR, estimated glomerular filtration rate; Tf:transferrin; GPX4:glutathione peroxidase 4; ACSL4, acyl-CoA synthetase long-chain family member 4.

### 3.6 Multinomial logistic regression analysis of the relationships between the severity of DN and serum GPX4, ACSL4, and iron metabolism indexes in T2DM patients

DN severity was considered as the dependent variable (NO=1, MI=2, MA=3), UA, SCr, TG, FINS, eGFR,GPX4, ACSL4, iron, Tf, and Ferritin as independent variables, multinomial logistic regression analysis was performed to evaluate the associations between DN severity and GPX4, ACSL4, iron, Tf, and Ferritin levels.

In Model 1, the NO and MI groups as reference groups, respectively; serum Tf and GPX4 were negatively correlated with DN severity in the MA group, and serum ACSL4 was positively correlated with DN severity in the MA group (**Table 4**). In Model 2, after adjusting for confounding factors such as UA, SCr, and eGFR based on Model 1, the NO and MI groups as reference groups, respectively. Serum Tf and GPX4 were still negatively correlated with DN severity in the MA group, and serum ACSL4 was positively correlated with DN severity in the MA group (**Table 4**). In Model 3, after adjusting for confounding factors such as TG and FINS based on Model 2, the NO and MI groups as reference groups, respectively. Serum Tf and GPX4 were still negatively correlated with DN severity in the MA group, and serum ACSL4 was positively correlated with DN severity in the MA group (**Table 4**).

**Table 4:** Multinomial logistic regression analysis of the relationship between the severity of DN and iron metabolism indexes, serum GPX4 levels, and serum ACSL4 levels in patients with T2DM.

Dependent variables	Independent variables	Model 1				Model 2				Model 3			
DN		β	Wald	OR(95%CI)	*P*	β	Wald	OR(95%CI)	*P*	β	Wald	OR(95%CI)	*P*
NO group ^a^	Iron	-0.025	0.276	0.975(0.888,1.071)	0.599	-0.073	1.477	0.930(0.827,1.046)	0.224	-0.075	1.543	0.908(0.824,1.044)	0.214
MI group	Tf	-0.184	0.106	0.832(0.276,2.510)	0.744	-0.143	0.039	0.867(0.210,3.572)	0.843	-0.096	0.017	0.908(0.213,3.866)	0.897
	Ferritin	-0.002	1.025	0.998(0.993,1.002)	0.311	-0.001	0.035	0.999(0.994,1.005)	0.852	0.001	0.013	1.000(0.994,1.005)	0.909
	GPX4	-0.012	0.672	0.988(0.960,1.017)	0.412	0.024	1.615	1.024(0.987,1.062)	0.204	0.024	1.659	1.025(0.987,1.064)	0.198
	ACSL4	0.085	0.833	1.089(0.907,1.308)	0.361	0.051	0.177	1.052(0.831,1.331)	0.674	0.046	0.141	1.047(0.825,1.328)	0.707
NO group ^a^	Iron	-0.027	0.145	0.973(0.846,1.119)	0.703	-0.38	0.224	0.963(0.823,1.126)	0.636	-0.039	0.237	0.961(0.821,1.126)	0.627
MA group	Tf	-1.802	4.692	0.165(0.032,0.842)	0.030	-2.19	4.155	0.112(0.014,0.919)	0.042	-2.221	4.081	0.109(0.013,0.936)	0.043
	Ferritin	-0.001	0.277	0.999(0.994,1.003))	0.599	-0.001	0.028	0.999(0.993,1.006)	0.866	-0.001	0.125	0.999(0.993,1.005)	0.723
	GPX4	-0.097	13.10	0.907(0.861,0.956)	<0.001	-0.078	5.661	0.925(0.867,0.986)	0.017	-0.078	5.897	0.925(0.868,0.985)	0.015
	ACSL4	0.630	19.91	1.878(1.424,2.477)	<0.001	0.684	16.156	1.983(1.420,2.768)	<0.001	0.669	14.60	1.952(1.385,2.751)	<0.001
MI group^a^	Iron	-0.002	0.070	0.998(0.870,1.145)	0.977	0.035	0.247	1.036(0.902,1.189)	0.619	0.036	0.253	1.036(0.902,1.191)	0.615
MA group	Tf	-1.619	4.168	0.198(0.042,0.937)	0.041	-2.047	4.441	0.129(0.019,0.867)	0.035	-2.125	4.493	0.119(0.017,0.852)	0.034
	Ferritin	0.001	0.198	1.001(0.996,1.006)	0.657	0.001	0.001	1.000(0.995,1.005)	0.995	-0.001	0.062	0.999(0.993,1.005)	0.803
	GPX4	-0.085	10.78	0.918(0.873,0.966)	0.001	-0.102	10.905	0.903(0.850,0.959)	0.001	-0.103	11.32	0.902(0.850,0.958)	0.001
	ACSL4	0.545	17.19	1.725(1.333,2.232)	<0.001	0.634	16.164	1.885(1.384,2.568)	<0.001	0.623	15.33	1.865(1.365,2.547)	<0.001

OR, odds ratio; CI, confidence interval. a:reference group.

Model 1, not adjusted; Model 2, adjusted Model 1+UA,SCr+eGFR; Model 3, adjusted Model 2+TG and FINS.

## 4 Discussion

Previous studies demonstrated that iron metabolism and ferroptosis are closely associated. In this study, we found that serum GPX4 was negatively correlated with serum ferritin and that ACSL4 was negatively correlated with serum iron and Tf, suggesting that ferritin and Tf may be involved in the development of ferroptosis, possibly because of the excessive deposition of iron and Tf in the kidney (8).

UAER is an important index of DN severity, and an increasing number of studies have shown that iron metabolism is associated with the occurrence and progression of DN (20).In the present study, we found that UAER and serum Tf levels were independently and negatively correlated, and serum Tf levels tended to decrease as the severity of DN increased. Similarly, Zhaoet al. reported that low Tf levels were associated with ERSD (8). Tf is a more sensitive indicator of glomerular injury than albumin and can be filtered more easily through the glomerular barrier (21).Decreased serum Tf levels in patients with DN may be attributed to the increased urinary excretion of Tf and increased Tf deposition in the kidneys (8).

Ferritin is a major iron storage protein involved in a wide range of physiological and pathological processes and is a serum marker of systemic iron stores (22). A positive correlation between serum ferritin and urinary microalbumin levels in patients with T2DM has been reported (9, 23). In the present study, serum ferritin was also found to be positively correlated with UAER. It is hypothesised that excessive iron stores may induce ROS release and promote oxidative stress; alternatively, ferritin may participate in ferroptosis via autophagy (24), thus promoting renal injury in patients with DN. Ferritin is also affected by inflammation and can disrupt glucose metabolism via the systemic inflammatory response, increasing the risk of diabetes mellitus and its complications (25).

Excessive iron is detrimental to health because the iron pool in cells can produce ROS *via*the Fenton reaction, which may be more pronounced in diabetes-induced, highly redox-active mitochondria (26). However, the present study demonstrated that serum iron levels were negatively correlated with UAER and decreased with increasing DN severity, which is inconsistent with the results of previous studies (3, 27). The possible reason is that the present study was conducted to detect serum iron levels, whereas previous studies were conducted to detect renal iron deposition in animals. The mechanism of decreased serum iron content is assumed to be related to excessive iron deposition in the kidneys on the one hand (8) and functional iron deficiency in patients on the other, which is characterised by the impaired release of stored iron (28).Iron deficiency anaemia has long been reported in DN (28).Similarly, Chen et al. showed that serum iron levels were lower in patients with DR than in non-DR patients (29).

A negative correlation between DN and serum GPX4 levels and a positive correlation between serum ACSL4 levels were observed in this study. Kim et al. also reported that ferroptosis was associated with DN, that ferroptosis-related molecules, such as SlC7A11 and GPX4, were reduced in kidney biopsy samples from patients with DN compared with those from non-DN patients, and that the mRNA and protein expression of SLC7A11 and GPX4 were reduced in the kidneys of DM mice compared with that in control mice (16).Wang et al. found that the expression of the ferroptosis-related protein GPX4 decreased, whereas ACSL4 expression increased; lipid peroxide products and iron content also increased in a DN mouse model (15).Tan et al. observed increased cell viability and apoptosis and decreased expression levels of GPX4 and SLC7A11 in NRK-52E cells induced by high glucose *in vitro*, suggesting that high glucose damages NRK-52E cells by triggering ferroptosis. *In vivo*, the expression of GPX4 and SLC7A11 was reduced in the kidneys of DM rats compared to that in control rats (30). MDA levels were increased, and SOD and GSH-Px levels were reduced in the kidneys of db/db mice than in the kidneys of db/m mice (31) To dated, the relationships between serum GPX4 and ACSL4 levels and DN in diabetic patients have not been reported. In the study, we found that the severity of DN was negatively correlated with serum GPX4 and positively correlated with ACSL4; however, the association between serum GPX4 and ACSL4 levels and expression levels of GPX4 and ACSL4 in renal tissue is not clear and further studies may be needed in the future.

A correlation between DN, ferroptosis, and iron metabolism was shown in this study, indicating that iron homeostasis imbalance and ferroptosis may be important steps in DN progression. DN pathogenesis includes altered renal haemodynamics, oxidative stress, inflammation, hypoxia, and the renin-angiotensin-aldosterone system (32). Iron metabolic homeostasis in diabetes patients is disrupted, with hyperglycaemia-induced ferrous iron overload and accumulation of ROS from multiple sources (3);moreover, the reduced utilisation of iron–binding sites in circulating Tf also promotes increased free iron-induced ROS accumulation (33), which depletes GPX4 content and promotes ferroptosis. Simultaneously, the disruption of system Xc function during ferroptosis prevents GPX4 from performing its normal antioxidant function (3), and activation of the Hippo pathway induces the upregulation of ACSL4 expression, leading to increased lipid peroxidation (34) and exacerbation of oxidative damage in the kidneys in DN; this may be a potential mechanism by which iron metabolism and ferroptosis promote the development and progression of DN. Interventions in ferroptosis may be a new direction to slow down the progression of DN.

Iron chelators are also effective in inhibiting ferroptosis and slowing the progression of DN by reducing excess intracellular iron, as the occurrence of ferroptosis depends on the generation of large amounts of ROS from excess intracellular iron *via* the Fenton reaction (35).The ACSL4 inhibitor rosiglitazone has been shown to markedly improve the survival of and renal function in DN mice and reduce renal MDA and iron levels (15).Huang et al. found that dapagliflozin improved ferroptosis in DN mice with tubular injury and that dagliflozin and SLC40A1 could bind to each other to reduce the ubiquitinated degradation of SLC40A1 and stabilise SLC40A1 to inhibit ferroptosis, thus improving tubular injury in DN mice (36). Empagliflozin prevents ferroptosis by activating the AMPK-NRF2 pathway (37). Glab ameliorated renal pathological changes and impaired renal function in DN mice, whereas it inhibited ferroptosis by increasing SOD and GSH activity and the expression of GPX4 and SLC7A11 and decreasing MDA and iron content and the expression of TFR1 (30). The ferroptosis inhibitor Fer-1 attenuates renal injury in DN mice (16, 31, 38). Li et al. reported that the treatment of DN mice with fenofibrate *in vitro* upregulated Nrf2 expression to inhibit diabetes-related ferroptosis and delay DN progression (3). Umbelliferone may inhibit ferroptosis,both *in vivo* and *in vitro*, by activating the Nrf2/HO-1 pathway to downregulate ACSL4 expression and upregulate GPX4 expression, eventually delaying DN (39).

Current studies on the relationship between ferroptosis markers and diabetic nephropathy are mostly limited to the cellular and animal levels, while diabetic nephropathy patients are not very well studied. The present study have found that serum ferroptosis markers GPX4 and ACSL4 as well as iron metabolism indexes are closely related to the severity of renal disease in type 2 diabetic patients, which may become biomarkers for diagnosis and treatment monitoring of diabetic nephropathy in the future. However, the present study has some limitations. First, this is a cross-sectional study and cannot elaborate on the causal relationship between DN and ferroptosis or iron metabolism; extensive cohort studies are needed to explore causality in the future. Second, the small number of participants may have led to a bias in the results. Third, due to the invasive nature of renal puncture biopsy, the diagnosis of DN is only based on clinical indicators and lacks pathological diagnosis via renal biopsy.

## 5 Conclusion

In conclusion, the results of this study showed that DN severity was negatively correlated with serum GPX4 and Tf levels and positively correlated with serum ACSL4 levels in patients with T2DM. The severity of DN correlates with ferroptosis and iron metabolism, which may serve as a guide for the early diagnosis, prevention, and delay of DN onset and progression.

## Data availability statement

The raw data supporting the conclusions of this article will be made available by the authors, without undue reservation.

## Ethics statement

The studies involving humans were approved by The First Hospital of Lanzhou University. The studies were conducted in accordance with the local legislation and institutional requirements. The human samples used in this study were acquired from primarily isolated as part of your previous study for which ethical approval was obtained. Written informed consent for participation was not required from the participants or the participants’ legal guardians/next of kin in accordance with the national legislation and institutional requirements.

## Author contributions

PZ: Methodology, Investigation, Software, Writing – original draft. XL: Writing – review & editing. ZZ: Investigation, Writing – review & editing. XY: Investigation, Writing – review & editing. YH: Investigation, Writing – review & editing. JL: Investigation, Writing – review & editing, Funding acquisition, Methodology.
